# Differential effects of clary sage (*Salvia sclarea* L.) oil and linalyl acetate on depression levels in diabetic foot ulcer patients with T2DM: a randomized blinded controlled trial

**DOI:** 10.3389/fmed.2025.1523441

**Published:** 2025-01-22

**Authors:** Ji Won Son, Purum Kang, Sun Seek Min, Geun Hee Seol

**Affiliations:** 1 Department of Basic Nursing Science, College of Nursing, Korea University, Seoul, Republic of Korea; 2 Department of Nursing, College of Nursing, Woosuk University, Jeonju, Republic of Korea; 3 Department of Physiology and Biophysics, Eulji University School of Medicine, Daejeon, Republic of Korea; 4 BK21 FOUR Program of Transdisciplinary Major in Learning Health Systems, Graduate School, Korea University, Seoul, Republic of Korea

**Keywords:** clary sage, linalyl acetate, diabetic foot ulcer, complementary and alternative medicine, depression

## Abstract

**Background:**

Diabetic foot ulcer (DFU) is a significant global health concern due to its substantial burden on individuals and healthcare systems. In addition to its physical impact, DFU has emotional effects on patients. This study analyzed the effects of inhaling clary sage (CS; *Salvia sclarea* L.) or linalyl acetate (LA) on depression and psychological factors in patients with DFU.

**Methods:**

This study, performed at the Diabetic Wound Center, enrolled DFU patients, categorized as having mild or moderate to severe depression based on visual analog scale (VAS) for depression scores. Patients were randomized to inhalation of 5% CS oil, 5% LA or almond oil (control) by natural breathing. Blood pressure, heart rate, Depression-VAS, Anxiety-VAS, and Stress-VAS were measured before and after inhalation.

**Results:**

This study enrolled 72 patients with DFU, including 43 with mild and 29 with moderate to severe depression. Of the 43 patients with mild depression, 14, 14, and 15 were randomized to inhalation of CS, LA, and almond oil, respectively. Of the 29 patients with moderate to severe depression, 9, 11, and 9 were randomized to inhalation of CS, LA, and almond oil, respectively. Compared with inhalation of almond oil, inhalation of CS oil significantly reduced stress (*p* < 0.05) and (*p* < 0.01) in patients with mild depression, whereas inhalation of LA significantly reduced anxiety-VAS (*p* < 0.05) and depression-VAS (*p* < 0.05) in patients with moderate to severe depression.

**Conclusion:**

Inhalation of CS oil may have the potential to alleviate stress and anxiety in DFU patients with mild depression, whereas inhalation of LA may have the potential to alleviate anxiety and depression in DFU patients with moderate to severe depression. These findings suggest that adjunct therapy in DFU patients should be individualized according to the degree of depression.

**Clinical trial registration:**

http://cris.nih.go.kr/, identifier KCT0009722.

## 1 Introduction

Diabetic foot ulcers (DFUs) are caused by poorly managed diabetes and have been associated with higher rates of foot amputation and mortality (1). DFUs can lead to deep tissue infections and various neurological abnormalities, with approximately 20% of affected patients requiring amputation (2). Quality of life was found to be significantly lower in patients with than without DFUs (3). Factors that reduce quality of life in DFU patients include prolonged treatment duration, difficulties in recovery, the need for strict glucose control, and resulting pain and mobility issues, all of which can negatively affect psychological well-being. Approximately 47% of patients with DFUs have been reported to exhibit symptoms of depression (4). Because anxiety and depression can worsen the condition of the ulcers, psychological support is also crucial for DFU patients (5). The 2023 guidelines published by the International Working Group on the Diabetic Foot (IWGDF) highlight the lack of research on psychological interventions for patients with DFUs (6), emphasizing the need for further research on both physiological and psychological treatments.

The use of complementary therapies involving essential oils has recently increased due to their effects and ease of access (7, 8). One of these essential oils, clary sage oil, has shown antidepressant and stress-relieving properties in several animal studies and clinical trials. For example, administration of 5% clary sage oil to rats via intraperitoneal injection or inhalation was found to affect dopaminergic pathways, demonstrating antidepressant and stress-relieving effects (9). Clary sage oil was also found to improve impaired endothelial function due to stress in rats by lowering blood corticosterone levels, indicating its potential for the prevention and treatment of cardiovascular diseases (10). Inhalation of clary sage oil was also reported to reduce pain and stress in patients with periodontal disease (11) and to reduce stress in women undergoing urodynamic testing (12).

The effects of clary sage oil are thought to be due to linalyl acetate, which constitutes the major component of clary sage (CS; *Salvia sclarea* L.) oil (12). Linalyl acetate has been reported to reduce systolic blood pressure and reactive oxygen species (ROS) production in a rat model of hypertension-related ischemic injury (13). In addition, linalyl acetate has been shown to exert drug-like effects by altering the expression of depression-related proteins (14), and to significantly reduce blood corticosterone levels in a rat model of type 2 diabetes induced by chronic stress and a high-fat diet, thereby alleviating stress (15). The ability of clary sage oil and linalyl acetate to affect psychological factors such as anxiety and depression, which have been reported to influence blood pressure and stress hormones, suggests that both clary sage oil and linalyl acetate may reduce the psychological burden and stress associated with managing chronic conditions such as DFU.

The present study therefore investigated the effects of clary sage oil and linalyl acetate on patients with type 2 diabetes and DFU. Because neuropsychological functions may be impaired in patients with severe depression, patients were categorized as having mild or moderate to severe depression and the effects of clary sage oil and linalyl acetate were analyzed in each group (16).

## 2 Methods

### 2.1 Study design and participants

This single-blind, randomized controlled trial was conducted from June 2023 to January 2024 with patients receiving care at a hospital in Korea. Patients were included if they (a) were aged ≥19 years, diagnosed with type 2 diabetes and receiving treatment for diabetic foot ulcers; (b) had not been diagnosed with olfactory disorders (sensorineural or conductive); (c) had orientation and communication abilities; (d) did not have allergic reactions to the essential oils included in this study; and (e) were not receiving medication for psychiatric disorders or hormone or essential oil therapy. Patients were excluded if they (a) were pregnant or breastfeeding; (b) were suspected of having systemic infections; (c) had mental disorders, drug or alcohol addiction, or were unable to understand the purpose and methods of this clinical trial; or (d) were deemed unsuitable for participation in the study by the researcher.

**Figure 1:**
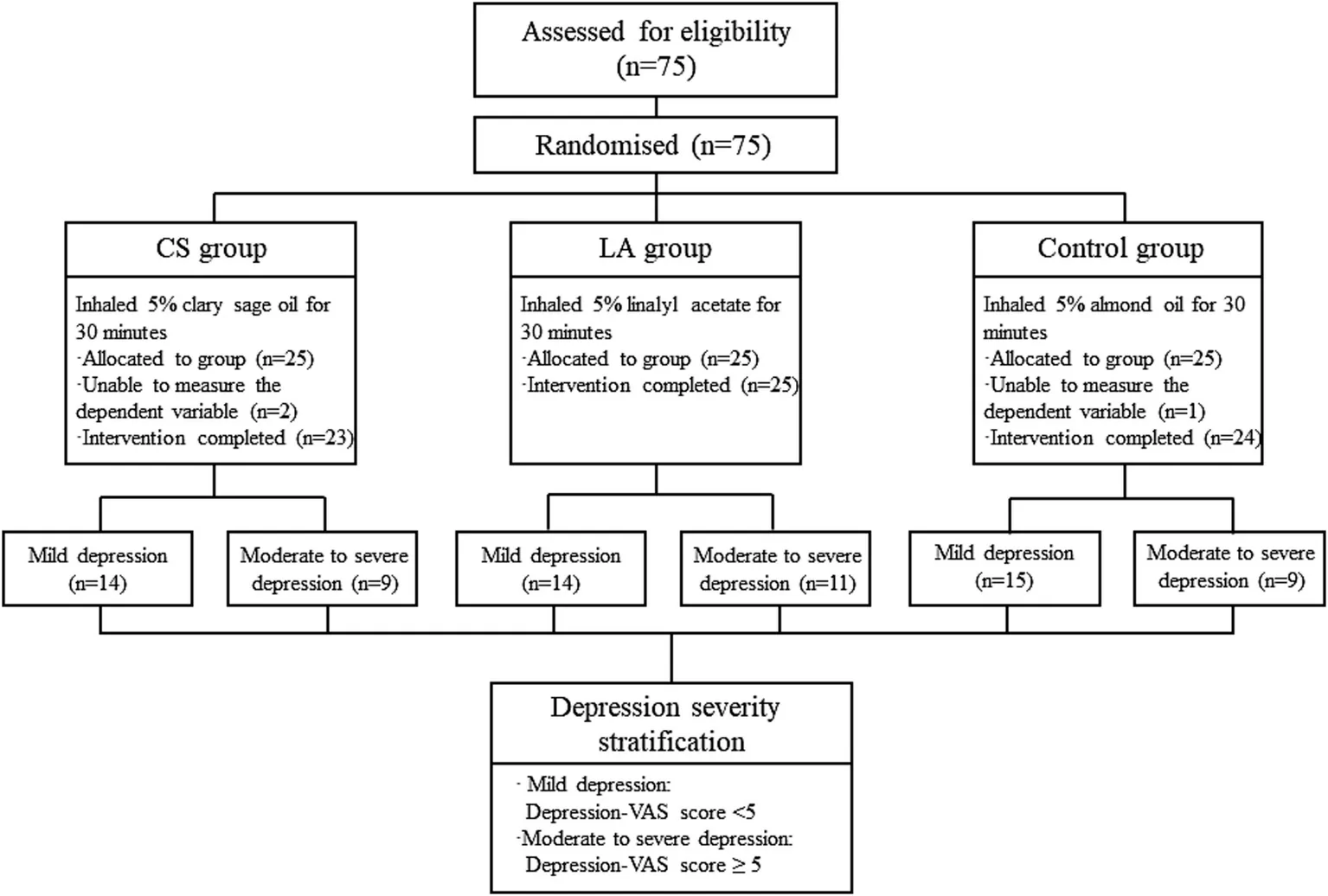
Recruitment and randomization. CS, clary sage; LA, linalyl acetate.

The minimum sample size for this study required to compare differences among the three groups was calculated by G*Power software (version 3.1) based on a previous study (17), with the significance level set at 0.05 and statistical power (1-*β*) at 0.8. This calculation estimated a minimum sample size of 22 participants per group, for a total of 66 participants. Based on an anticipated dropout rate of approximately 10%, 25 participants were assigned to each group. Ultimately, 3 participants were excluded due to the inability to accurately measure the dependent variables, resulting in a final sample of 72 participants (Figure 1). The study protocol was approved by the institutional review board of Korea University Guro Hospital (2023GR0197), and the study was retrospectively registered on the Clinical Research Information Service (Registration number, KCT0009722; initial registration 23 August 2024).

### 2.2 Gas chromatography–mass spectrometry profiling of clary sage oil

Clary sage oil was purchased from Aromarant Co. (Röttingen, Germany), and its composition was analyzed by gas chromatography–mass spectrometry (GC–MS). GC–MS analysis was developed in DB-WAXETR type capillary column (60 m × 0.25 mm i.d., 0.25 μm film thickness) provided by Agilent Technologies. The carrier gas was helium, maintained at the flow rate of 1.0 mL/min. The initial column temperature was 40°C, increasing 5°C/min to a maximum of 250°C. Phytochemical compounds were identified by their retention time, and were confirmed using reference samples.

### 2.3 Randomization and intervention

Before the start of this study, a pilot test was performed on five healthy adults to determine the optimal concentrations of clary sage oil and linalyl acetate in almond oil that would maintain the fragrance throughout the intervention period without causing discomfort. Based on previous studies, concentrations of 1, 5, and 10% were tested. Considering feedback indicating that the fragrance was not overwhelming, remained perceptible throughout the intervention, and caused no adverse effects, the concentration of essential oil for the intervention was set at 5%.

Participants who met the inclusion criteria were provided with a detailed explanation of the study’s purpose and procedures, after which written informed consent was obtained. An internal researcher was responsible for participant recruitment, screening and intervention, whereas external researchers prepared the oils and analyzed the data. Participants were randomized to three groups using a simple randomization sequence generated by Random Allocation Software (version 2.0). The sequence was generated by an external researcher and was concealed from the internal researcher. One group of subjects was treated with 5% clary sage oil diluted in almond oil; a second group was treated with 5% linalyl acetate diluted in almond oil; and the third (control group) was treated with almond oil. The three preparations were sealed in identical tubes, each labeled with a small number. The samples were prepared by an external researcher, who provided them to the internal research team.

Individuals who consented to participate in the study were asked to complete a questionnaire assessing their levels of pain, depression, anxiety, and stress. Blood pressure and heart rate were subsequently measured while participants were in the supine position. A 0.1 mL aliquot of essential oil was applied to a 1 cm x 2 cm gauze, which was affixed to the philtrum using skin tape. Participants were instructed to inhale naturally for 30 min, after which their blood pressure and heart rate were measured in the supine position. Participants subsequently completed a second questionnaire assessing their levels of pain, depression, anxiety, and stress.

### 2.4 Data collection

General characteristics such as gender, age, and medical history were collected. Depression, anxiety, and stress levels were assessed using visual analog scales (VAS). Each scale consisted of a 10 cm horizontal line, with 0 at the left end (indicating no depression, anxiety or stress) and 10 at the right end (indicating severe depression, anxiety or stress). Participants marked the point that best represented their perceived levels of depression, anxiety, and stress. Blood pressure and heart rate were measured after a 5-min rest period using an electronic sphygmomanometer (CF155f, Rossmax, Taiwan) on the right brachial artery (or the left brachial artery for patients with an arteriovenous fistula), with each measured once before and once after the aromatherapy intervention.

### 2.5 Statistical analysis

Descriptive statistics were presented as mean ± standard deviation (SD) or as number (percentage). Categorical variables were analyzed using chi-square tests, and continuous variables were analyzed using Shapiro–Wilk tests. Normally distributed data in the three groups were analyzed by one-way analysis of variance (ANOVA), and non-normally distributed data were analyzed by Kruskal-Wallis tests. Differences before and after inhalation within each group were analyzed using paired t-tests or Wilcoxon signed-rank tests, depending on data normality. All statistical analyses were performed using SPSS version 27.0 statistical software (IBM Inc., Armonk, NY, USA), with *p*-values <0.05 considered statistically significant.

## 3 Results

### 3.1 Chemical composition of clary sage oil

**Figure 2:**
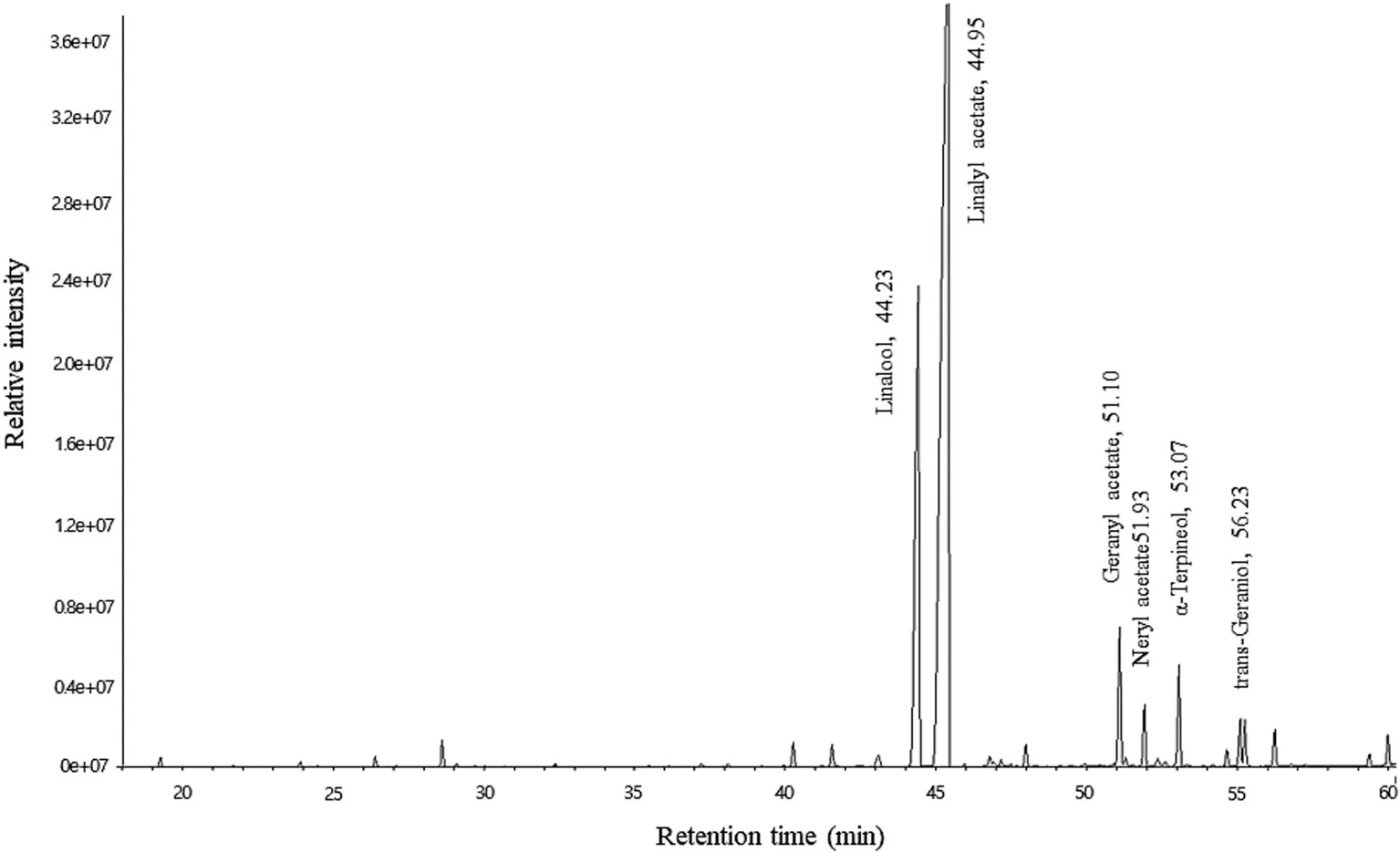
Total ion chromatogram of clary sage oil derived from gas chromatography–mass spectrometry data.

The total ion chromatogram of clary sage oil reveals the presence of these compounds, as shown in Figure 2. Two major compounds of clary sage oil were linalyl acetate (71.2%) and linalool (16.2%), followed by geranyl acetate (3.43%), *α*-terpineol (2.95%), neryl acetate (1.68%) and trans-geraniol (1.68%).

### 3.2 Baseline characteristics

The 72 participants included 49 (68.1%) male and 23 (31.9%) female, with a mean age of 67.99 ± 9.42 years and a mean body mass index (BMI) of 23.08 ± 2.90 kg/m^2^. Of the 72 participants, 54 (75%) had been diagnosed with hypertension and 40 (55.6%) with chronic kidney disease. Gender distribution, age, obesity, and the presence of hypertension did not differ significantly among the three groups (Table 1). The percentage of subjects with chronic kidney disease was significantly higher in the control group (18/24; 75.0%) than in the clary sage (CS) oil (9/23; 39.1%) and linalyl acetate (LA) (13/25; 52%) groups (*p* = 0.043). In contrast, levels of depression, anxiety, stress (as measured by VAS), as well as systolic blood pressure and heart rate, did not differ significantly in the three groups.

**Table 1:** Baseline characteristics.

Variable	Total (*n* = 72)	Group I (*n* = 23)	Group II (*n* = 25)	Group III (*n* = 24)	*p-*value
Gender, *n* (%)					0.442
Male	49 (68.1)	18 (78.3)	16 (64.0)	15 (62.5)	
Female	23 (31.9)	5 (25.0)	9 (36.0)	9 (37.5)	
Age (years)	67.99 ± 9.42	69.26 ± 9.57	67.12 ± 10.17	67.67 ± 8.72	0.725
Body mass index (kg/m^2^)	23.08 ± 2.90	23.23 ± 2.33	23.30 ± 2.67	22.71 ± 3.62	0.732^a^
Hypertension, *n* (%)					0.706
Yes	54 (75.0)	16 (69.6)	20 (80.0)	18 (75.0)	
No	18 (25.0)	7 (30.4)	5 (20.0)	6 (25.0)	
Chronic kidney disease, *n* (%)					<0.05*
Yes	40 (55.6)	9 (39.1)	13 (52.0)	18 (75.0)	
No	32 (44.4)	14 (60.9)	12 (48.0)	6 (25.0)	
Heart rate (bpm)	76.79 ± 13.22	75.87 ± 13.09	78.24 ± 14.83	76.17 ± 11.93	0.797
Systolic blood pressure (mmHg)	136.90 ± 23.75	135.26 ± 24.49	138.24 ± 24.56	137.08 ± 23.09	0.911
Diastolic blood pressure (mmHg)	74.85 ± 11.46	76.83 ± 10.27	77.68 ± 12.38	70.00 ± 10.36	<0.05*^a^
Mean arterial pressure (mmHg)	95.53 ± 13.67	96.30 ± 13.05	97.87 ± 15.27	92.36 ± 12.40	0.356
Depression-VAS (cm)	3.86 ± 2.67	3.64 ± 2.63	4.02 ± 2.70	3.92 ± 2.80	0.884
Anxiety-VAS (cm)	4.53 ± 2.81	4.64 ± 2.21	4.42 ± 2.75	4.53 ± 3.44	0.953^a^
Stress-VAS (cm)	3.87 ± 2.62	3.73 ± 2.23	3.83 ± 2.57	4.04 ± 3.09	0.921

Group I: Inhaled 5% clary sage oil for 30 min, Group II: inhaled 5% linalyl acetate for 30 min, Group III: inhaled almond oil for 30 min.

Data are expressed as mean ± SD. VAS, Visual Analog Scale.

a Analyzed using the Kruskal-Wallis test. ^*^p < 0.05.

### 3.3 Effects of clary sage oil and linalyl acetate inhalation

Comparisons of the three groups after inhalation showed a significant difference in mean arterial pressure between the LA and control groups (*p* < 0.05, Table 2). Post-hoc analysis using the Mann–Whitney *U* test confirmed a significant difference between these two groups (*p* < 0.05).

**Table 2:** Effects of Clary Sage oil and Linalyl Acetate.

Variable	Group I (*n* = 23)			Group II (*n* = 25)			Group III (*n* = 24)			*p*-value (between-group)
	Pre	Post	*p*-value (within)	Pre	Post	*p*-value (within)	Pre	Post	*p*-value (within)	
Depression-VAS (cm)	3.64 ± 2.63	3.30 ± 2.79	0.310	4.02 ± 2.70	3.04 ± 2.48	0.041^*^	3.92 ± 2.80	3.87 ± 2.74	0.838	0.255^b^
Anxiety-VAS (cm)	4.64 ± 2.21	3.31 ± 2.66	<0.05^*^	4.42 ± 2.75	2.99 ± 1.99	<0.05^*^	4.53 ± 3.44	4.25 ± 2.77	0.469^a^	0.288^b^
Stress-VAS (cm)	3.73 ± 2.23	3.18 ± 2.67	0.300	3.83 ± 2.57	3.09 ± 2.37	0.107	4.04 ± 3.09	4.04 ± 3.16	1.000	0.348^b^
SBP (mmHg)	135.26 ± 24.49	132.00 ± 22.67	0.302	138.24 ± 24.56	137.76 ± 21.75	0.875	137.08 ± 23.09	141.08 ± 23.67	0.063	0.207^b^
MAP (mmHg)	96.30 ± 13.05	94.32 ± 12.59	0.138	97.87 ± 15.27	95.28 ± 14.10	0.161	92.36 ± 12.40	94.39 ± 11.69	0.097	<0.05^*^^b^
Heart rate (bpm)	75.87 ± 13.09	73.74 ± 13.02	<0.05^*^	78.24 ± 14.83	76.84 ± 14.62	0.272	76.17 ± 11.93	75.50 ± 12.50	0.175	0.112^b^

Group I: Inhaled 5% clary sage oil for 30 min, Group II: inhaled 5% linalyl acetate for 30 min, Group III: inhaled almond oil for 30 min. Data are expressed as mean ± SD. VAS, Visual Analog Scale; SBP, Systolic Blood Pressure; MAP, Mean Arterial Pressure.

a Analyzed using the Wilcoxon signed-rank test.

b Analyzed using the Kruskal-Wallis test. ^*^p < 0.05.

Comparisons before and after intervention showed significant reductions in anxiety (*p* < 0.05) and heart rate (*p* < 0.05) in the CS group and significant reductions in depression (*p* < 0.05) and anxiety (*p* < 0.05) in the LA group. In contrast, there were no significant differences from before to after intervention in the control group.

### 3.4 Baseline characteristics of participants as a function of patient-reported depression severity

Based on the patient-reported depression VAS scores, scores below 5 were classified as mild depression, while scores of 5 or higher were classified as moderate to severe depression. Of the 72 patients, 43 (59.7%) had mild depression and 29 (40.3%) had moderate to severe depression, with mean (SD) depression-VAS scores of 2.03 ± 1.44 and 6.59 ± 1.48, respectively (*p* < 0.001). There were no significant differences between these two subgroups in age, BMI, presence of chronic kidney disease, presence of hypertension, systolic blood pressure, mean arterial pressure and heart rate. However, anxiety-VAS scores (*p* < 0.001) and stress-VAS scores (*p* < 0.001) were significantly higher in patients with moderate to severe depression than in patients with mild depression (Table 3).

**Table 3:** Baseline characteristics of patient with Diabetic foot (DMF) as patient-reported depression.

Variable		Depression-VAS (cm)		*p* value
		< 5 (*n* = 43)	≥ 5 (*n* = 29)	
Gender, *n* (%)	Male	32 (74.4)	17 (58.6)	0.159
	Female	11 (25.6)	12 (41.4)	
Age (years)		67.40 ± 8.96	68.86 ± 10.18	0.521
BMI (kg/m^2^)		23.37 ± 3.19	22.65 ± 2.40	0.307
Hypertension, *n* (%)	Yes	32 (74.4)	22 (75.9)	0.890
	No	11 (25.6)	7 (24.1)	
CKD	Yes	21 (48.8)	19 (65.5)	0.162
	No	22 (51.2)	10 (34.5)	
SBP (mmHg)		132.79 ± 22.07	143.00 ± 25.20	0.073
DBP (mmHg)		75.42 ± 10.25	74.00 ± 13.19	0.610
MAP (mmHg)		94.54 ± 12.36	97.00 ± 15.53	0.458
HR (bpm)		77.40 ± 13.08	75.90 ± 13.60	0.640
Anxiety-VAS (cm)		3.52 ± 2.61	6.02 ± 2.45	<0.001^a^
Stress-VAS (cm)		2.69 ± 2.15	5.62 ± 2.28	<0.001^a^
Depression-VAS (cm)		2.03 ± 1.44	6.59 ± 1.48	<0.001^a^

Data are expressed as mean ± SD. BMI, body mass index; CKD, chronic kidney disease; SBP, systolic blood pressure; DBP, diastolic blood pressure; MAP, mean arterial pressure; HR, heart rate; VAS, visual analog scale.

a *p*-value calculated by Mann–Whitney test.

### 3.5 Effects of clary sage oil and linalyl acetate inhalation as a function of patient-reported depression severity

**Figure 3:**
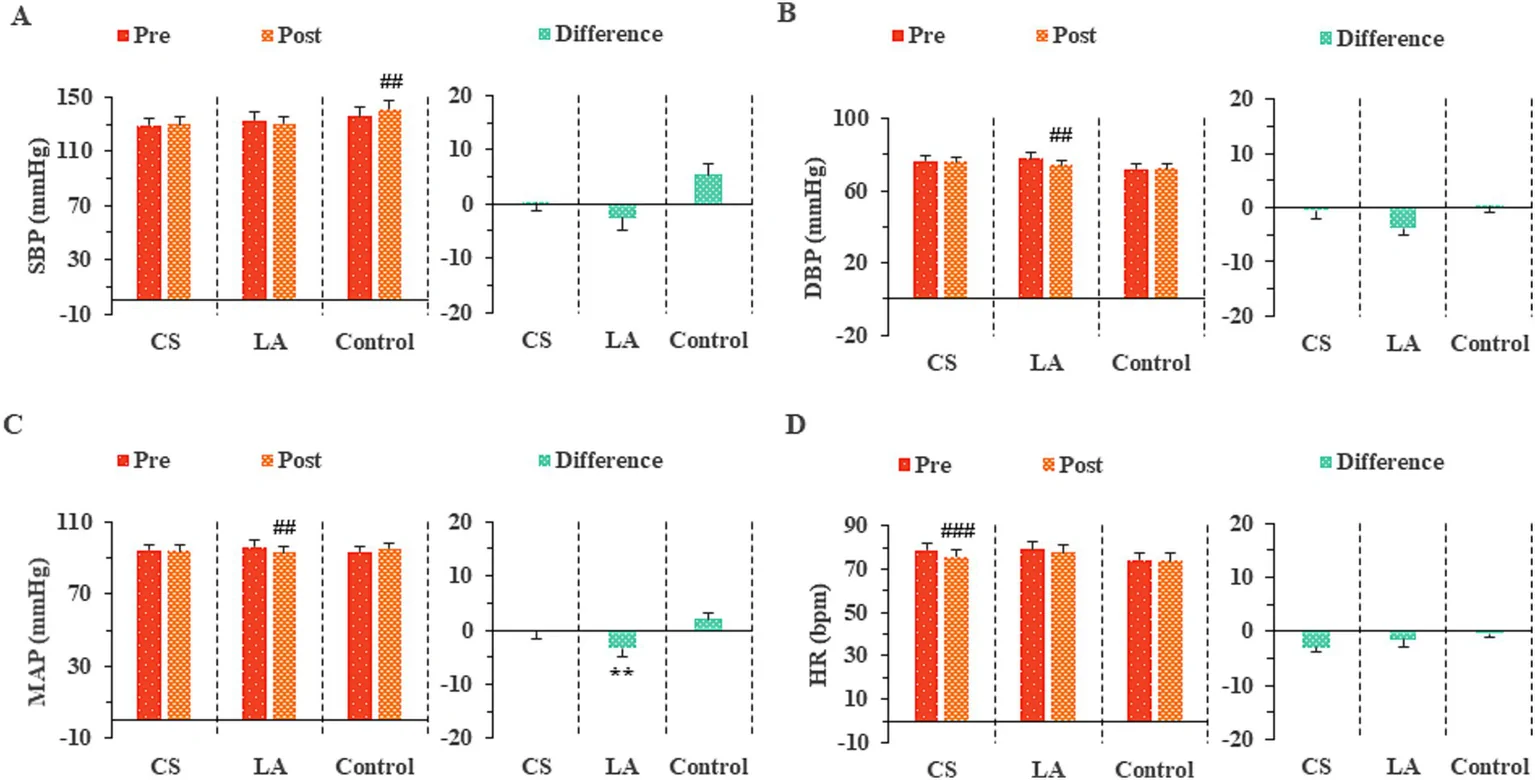
Effects of inhalation of clary sage oil (CS) or linalyl acetate (LA) on **(A)** systolic blood pressure (SBP), **(B)** diastolic blood pressure (DBP), **(C)** mean arterial pressure (MAP), and **(D)** heart rate (HR) in DFU patients with mild depression. Results are presented as mean ± standard error of the mean. ##*p* < 0.05, ###*p* < 0.01 vs. pretreatment; **p < 0.05 vs. Control.

**Figure 4:**
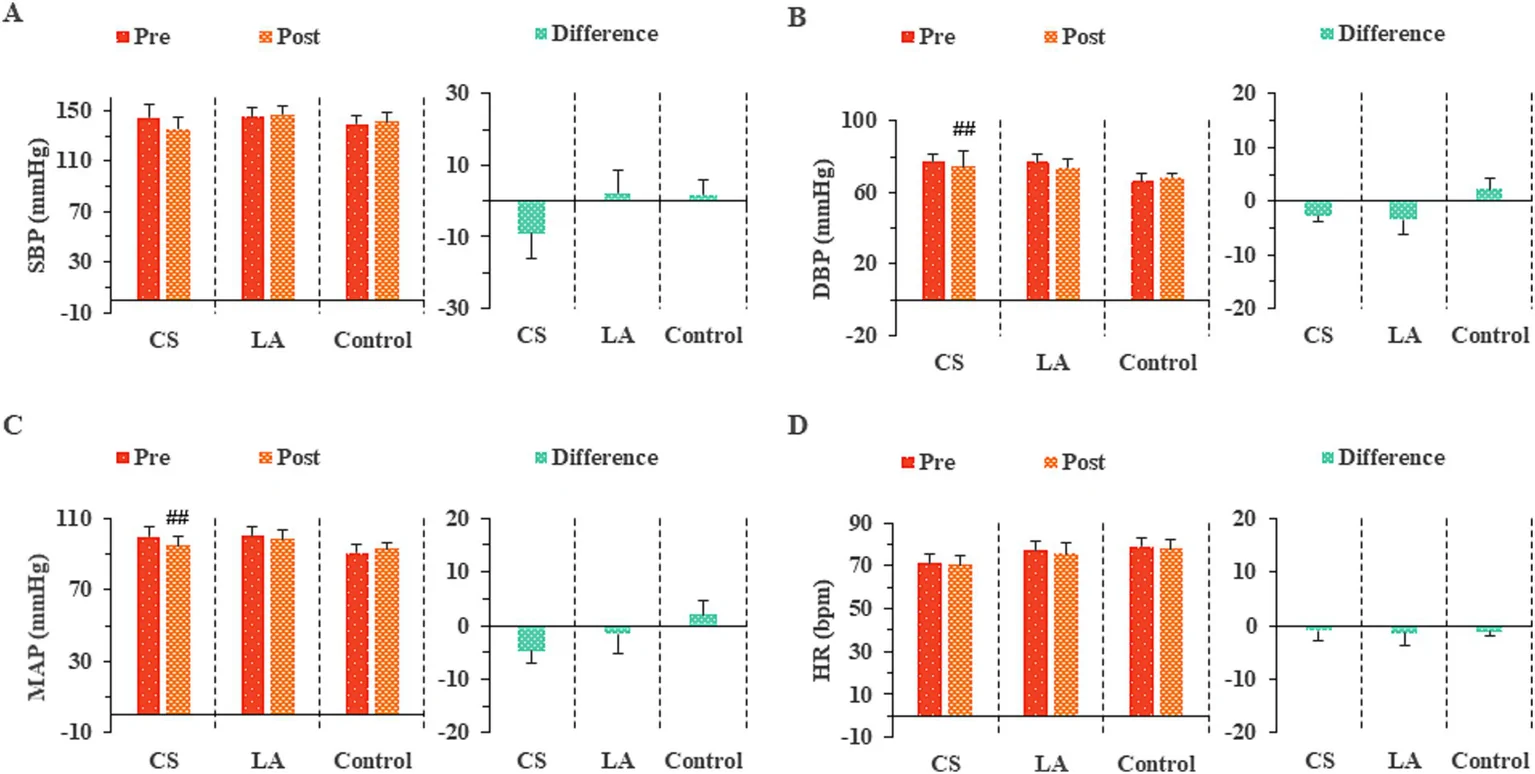
Effects of inhalation of clary sage oil (CS) or linalyl acetate (LA) on **(A)** systolic blood pressure (SBP), **(B)** diastolic blood pressure (DBP), **(C)** mean arterial pressure (MAP), and **(D)** heart rate (HR) in DFU patients with moderate to severe high depression. Results are presented as mean ± standard error of the mean. ##*p* < 0.05 vs. pretreatment.

The effects of clary sage oil and linalyl acetate inhalation on anxiety and stress were separately analyzed in subjects with mild and moderate to severe depression. Inhalation of clary sage oil significantly reduced heart rate (*p* < 0.01) in patients with mild depression, whereas inhalation of linalyl acetate significantly reduced diastolic blood pressure (*p* < 0.05) and mean arterial pressure (*p* < 0.05) in patients with moderate to severe depression (Figure 3); the reduction of mean arterial pressure in this group was also significantly greater than in the control group (*p* < 0.05). Although inhalation of clary sage oil significantly reduced diastolic blood pressure (*p* < 0.05) in patients with moderate to severe depression (Figure 4), diastolic blood pressure did not differ significantly among the three subgroups. Relative to baseline, inhalation of clary sage oil significantly reduced anxiety-VAS (*p* < 0.01) and stress-VAS (*p* < 0.05) in patients with mild depression, with the reduction in anxiety-VAS being significantly greater in the clary sage oil than in the control group (*p* < 0.05, Table 4). In contrast, inhalation of linalyl acetate significantly reduced anxiety-VAS (*p* < 0.05) and depression-VAS (*p* < 0.05) relative to baseline in patients with moderate to severe depression. Psychological status, however, did not differ significantly among the three subgroups of patients with mild and moderate to severe depression (Table 4).

**Table 4:** Clinical characteristics at pre and post intervention in groups.

Variable	Depression-VAS (cm) < 5 (*n* = 43)			*p* value	Depression-VAS (cm) ≥ 5 (n = 29)			*p* value
	Group I (*n* = 14)	Group II (*n* = 14)	Group III (*n* = 15)		Group I (*n* = 9)	Group II (*n* = 11)	Group III (*n* = 9)	
Anxiety-VAS (cm)								
Pre	4.06 ± 2.37	3.35 ± 2.66	3.19 ± 2.85		5.54 ± 1.66	5.78 ± 2.29	6.78 ± 3.27	
Post	1.91 ± 1.62	2.51 ± 1.87	3.27 ± 2.60		5.48 ± 2.53	3.60 ± 2.06	5.89 ± 2.32	
Mean difference	−2.14 ± 2.47	−0.84 ± 3.22	0.08 ± 1.36	<0.05^*^^b^	−0.07 ± 2.50	−2.18 ± 2.52	−0.89 ± 2.47	0.160^b^
*p*-value	<0.01^*^	0.350	0.779^a^		0.938	<0.05^*^^a^	0.312	
Stress-VAS (cm)								
Pre	3.00 ± 1.96	2.61 ± 2.31	2.46 ± 2.27		4.87 ± 2.24	5.38 ± 2.05	6.67 ± 2.45	
Post	1.77 ± 1.36	2.01 ± 2.05	2.53 ± 2.33		5.37 ± 2.78	4.46 ± 2.06	6.56 ± 2.79	
Mean difference	−1.23 ± 1.83	−0.61 ± 2.59	0.07 ± 0.88	0.142^b^	−0.50 ± 3.10	−0.92 ± 1.75	−0.11 ± 1.05	0.286^b^
*p*-value	<0.05^*^	0.397	0.783^a^		0.642	0.112	0.785^a^	
Depression-VAS (cm)								
Pre	1.90 ± 1.55	2.04 ± 1.40	2.13 ± 1.45		6.34 ± 1.24	6.54 ± 1.56	6.89 ± 1.69	
Post	1.62 ± 1.39	1.91 ± 2.05	2.39 ± 1.92	0.887^b^	5.91 ± 2.38	4.48 ± 2.28	6.33 ± 2.06	0.124^b^
Mean difference	−0.28 ± 1.10	−0.13 ± 1.69	0.26 ± 0.96		−0.43 ± 2.18	−2.05 ± 2.49	−0.56 ± 1.13	
*p*-value	0.553^a^	0.781	0.395^a^		0.568	<0.05^*^	0.180	

Group I: Inhaled 5% clary sage oil for 30 min, Group II: inhaled 5% linalyl acetate for 30 min, Group III: inhaled almond oil for 30 min.

Data are expressed as mean ± SD. VAS, visual analog scale.

Values are presented as mean ± standard deviation. *p*-values were calculated using paired *t*-test. * *p* < 0.05.

a *p*-value calculated by Wilcoxon signed-rank test.

b *p*-value calculated by Kruskal-Wallis test.

## 4 Discussion

This study explored the effects of clary sage oil and its main component, linalyl acetate, on depression, anxiety, blood pressure, and heart rate in patients with DFU who had been diagnosed with type 2 diabetes. Inhalation of clary sage oil was found to reduce self-reported depression level and heart rate in these patients, whereas linalyl acetate was found to reduce self-reported depression and anxiety levels, as well as blood pressure, in type 2 diabetes patients with DFU. When patients were subgrouped by levels of depression, clary sage oil was found effective in reducing anxiety and stress in patients with mild depression, whereas linalyl acetate was more effective in alleviating anxiety in patients with moderate to severe depression.

The mean arterial pressure was found to be significantly lower in subjects who inhaled 5% linalyl acetate than in those who inhaled 5% clary sage oil or almond oil alone. In contrast, although inhalation of clary sage oil tended to reduce mean arterial pressure, the reduction did not differ significantly compared with the other groups. Similarly, a previous study found no statistically significant differences between inhalation of clary sage oil and control subjects, although blood pressure tended to be lower (18). Clary sage oil has been found to alleviate anxiety and reduce stress (19, 20), responses likely mediated through the regulation of the autonomic nervous system (21). The significant effect observed in participants who inhaled linalyl acetate suggests that clary sage oil’s effects may largely be attributed to linalyl acetate, which has been found to reduce blood pressure (13). However, the proportion of CKD patients was higher in the control group, although baseline blood pressure levels were not elevated, and no between-group differences were observed in psychological variables. These findings suggest that the results remain valid despite the imbalance in CKD prevalence, indicating that the conclusions remain meaningful within the context of this study.

This study also found that linalyl acetate inhalation significantly reduced mean arterial pressure in participants with mild depression. Moreover, inhalation of clary sage oil was found to significantly reduce mean arterial pressure in subjects with moderate to severe depression. While the observed differences in efficacy between linalyl acetate and clary sage oil may be partially attributed to other components of clary sage oil, the observed effects are driven in large part by linalyl acetate. Clary sage oil has been shown to influence dopaminergic pathways and may be involved in sympathetic nervous system responses (9). Because stress responses are affected by catecholamine reactions associated with stress (22), the effects of clary sage oil on blood pressure may be mediated by its ability to regulate stress experienced during the treatment process in patients with DFU. Additionally, linalyl acetate has been found to act directly on vascular smooth muscle, inducing vasodilation and thereby lowering blood pressure (23). This vasodilatory effect may be mediated by enhancement of nitric oxide signaling pathways, which are known to play a crucial role in vascular smooth muscle relaxation. In addition to its vascular actions, linalyl acetate has been shown to enhance parasympathetic nervous system activity, reducing stress and anxiety (19). This effect may also involve the modulation of serotonin and dopamine receptors, further contributing to the ability of linalyl acetate to regulate stress and emotional responses (14). Additionally, linalyl acetate is known to increase endothelial nitric oxide synthase expression, promoting vasodilation and providing cardiovascular benefits, particularly in individuals with compromised cardiovascular function (13). Considering that all participants in the present study were in the prehypertensive stage, these cardiovascular effects likely contributed to the observed reductions in blood pressure and improved outcomes. Collectively, these findings suggest that while the effects of clary sage oil may vary slightly due to its complex composition, linalyl acetate plays a predominant role in the cardiovascular and autonomic benefits conferred by clary sage oil. These findings suggest that clary sage oil and linalyl acetate not only regulate the emotional responses triggered by stress during wound care but also modulate autonomic nervous system responses to reduce blood pressure. Additionally, their effects may partly be attributed to direct vasodilation and improved cardiovascular function.

The results of this study align with previous findings that inhalation of clary sage oil or linalyl acetate has anxiolytic effects in patients undergoing cancer treatment (19). The ability of clary sage oil to significantly reduce heart rate within normal ranges in patients with lower levels of depression likely involves activation of the parasympathetic nervous system. This may have been due to the anxiolytic properties of clary sage oil, with these results being consistent with findings from studies involving clary sage oil administration to rats (10) and healthy individuals (24). However, the absence of such effects in subjects administered linalyl acetate suggests that other components of clary sage oil may contribute to these outcomes. In contrast, linalyl acetate exhibited both anxiolytic and antidepressant effects in patients with higher levels of depression. These findings were consistent with results in hemodialysis patients (25) and elderly community-dwelling individuals (26), who experienced antidepressant benefits from inhaling lavender oil, which also contains linalyl acetate as a primary component. Because more severe infections in DFU patients have been associated with significantly higher levels of depression (27), effective psychological interventions are critical for those with higher levels of depression. Thus, linalyl acetate inhalation may have psychological benefits, particularly in DFU patients with severe infections and/or higher levels of depression.

The present study also found that clary sage oil was effective in reducing anxiety and stress in patients with lower levels of depression, whereas linalyl acetate was effective in alleviating anxiety and depression in patients with higher levels of depression. This result provides an evidence-based foundation for the use of clary sage oil and its main component, linalyl acetate, as non-pharmacological psychological interventions for patients with DFU. Furthermore, these results suggest the importance of assessing the level of depression in these patients and providing tailored interventions accordingly.

This study has several limitations. First, it was conducted at a single institution, which may limit the generalizability of the findings to broader populations. Second, the proportion of CKD patients was higher in the control group, necessitating caution when interpreting the results. Lastly, the study focused only on the short-term effects of the interventions, leaving the long-term impacts unexplored. Therefore, future studies should be undertaken that incorporate a multi-center design to enhance the generalizability of the findings and that conduct long-term follow-up to evaluate the sustained effects of clary sage oil and linalyl acetate. Additionally, future studies should seek to better balance the baseline characteristics, such as the proportion of CKD patients, across groups to improve the validity and reliability of the results.

## Data Availability

The raw data supporting the conclusions of this article will be made available by the authors, without undue reservation.
